# Nanovaccines to Combat *Aeromonas hydrophila* Infections in Warm-Water Aquaculture: Opportunities and Challenges

**DOI:** 10.3390/vaccines11101555

**Published:** 2023-10-01

**Authors:** Mave Harshitha, Ashwath Nayak, Somanath Disha, Uchangi Satyaprasad Akshath, Saurabh Dubey, Hetron Mweemba Munang’andu, Anirban Chakraborty, Indrani Karunasagar, Biswajit Maiti

**Affiliations:** 1 Nitte (Deemed to be University), Nitte University Centre for Science Education and Research, Department of Bio & Nano Technology, Paneer Campus, Deralakatte, Mangalore 575018, Indiadisha.20phdbs103@student.nitte.edu.in (S.D.); akshath.uchangi@nitte.edu.in (U.S.A.); 2 Section of Experimental Biomedicine, Department of Production Animal Clinical Sciences, Faculty of Veterinary Medicine, Norwegian University of Life Sciences, P.O. Box 5003, N-1432 Ås, Norway; saurabh.dubey@nmbu.no; 3 Department of Biosciences and Aquaculture, Nord University, PB 1490, 8049 Bodø, Norway; hetron.m.munangandu@nord.no; 4 Nitte (Deemed to be University), Nitte University Centre for Science Education and Research, Department of Molecular Genetics & Cancer, Paneer Campus, Deralakatte, Mangaluru 575018, India; anirban@nitte.edu.in; 5 Nitte (Deemed to be University), DST Technology Enabling Centre, Paneer Campus, Deralakatte, Mangaluru 575018, India

**Keywords:** nanovaccines, vaccination, fish, aquaculture, *Aeromonas hydrophila*

## Abstract

The application of nanotechnology in aquaculture for developing efficient vaccines has shown great potential in recent years. Nanovaccination, which involves encapsulating antigens of fish pathogens in various polymeric materials and nanoparticles, can afford protection to the antigens and a sustained release of the molecule. Oral administration of nanoparticles would be a convenient and cost-effective method for delivering vaccines in aquaculture while eliminating the need for stressful, labour-intensive injectables. The small size of nanoparticles allows them to overcome the degradative digestive enzymes and help deliver antigens to the target site of the fish more effectively. This targeted-delivery approach would help trigger cellular and humoral immune responses more efficiently, thereby enhancing the protective efficacy of vaccines. This is particularly relevant for combating diseases caused by pathogens like *Aeromonas hydrophila*, a major fish pathogen responsible for significant morbidity and mortality in the aquaculture sector. While the use of nanoparticle-based vaccines in aquaculture has shown promise, concerns exist about the potential toxicity associated with certain types of nanoparticles. Some nanoparticles have been found to exhibit varying degrees of toxicity, and their safety profiles need to be thoroughly assessed before widespread application. The introduction of nanovaccines has opened new vistas for improving aquaculture healthcare, but must be evaluated for potential toxicity before aquaculture applications. Details of nanovaccines and their mode of action, with a focus on protecting fish from infections and outbreaks caused by the ubiquitous opportunistic pathogen *A. hydrophila*, are reviewed here.

## 1. Introduction

Aquaculture is a rapidly growing sector, serving as a vital source of easily digestible protein, useful lipids, and an array of essential micronutrients for addressing food security [1]. It is practiced globally and contributes significantly to countries’ gross domestic product [2]. According to recent data [3], global aquaculture production reached a record 122.6 million tonnes in 2020. The sector has emerged as a major source of revenue at both household and national levels in both developed and developing countries [4]. Globally, aquaculture is growing at a rate of 6% per annum. In 2017, global per capita fish consumption was approximately 20.3 kg, reflecting the demand for fish and fishery products due to their nutritional benefits [5] and is evidence that many individuals are engaged in fisheries. According to 2017 data, approximately 59.7 million people were engaged in fish-related professions, including capture and culture, with capture fishery production recording a steady increase over the years, and culture fishery production witnessing an exponential growth from 1 million tonnes in 1956 to 82 million tonnes in 2018 [6,7].

This high demand for fish and fisheries products puts strain on production systems, leading to higher stocking rates and increased stress on fish coupled with factors like overexploitation, urbanization, and pollution from untreated industrial waste. Consequently, the sector has witnessed the emergence of infectious diseases in cultured fish populations. Bacterial diseases, caused by pathogens such as *Aeromonas*, *Edwardsiella*, *Flavobacterium*, *Streptococcus*, and *Pseudomonas*, pose a significant threat and can lead to outbreaks and economic losses in aquaculture. *A. hydrophila*, *A. caviae*, and *A. veronii* are major opportunistic fish pathogens belonging to the *Aeromonadaceae* family, affecting warm-water aquaculture worldwide [8]. In 2009, epidemic outbreaks and significant economic losses were reported in catfish farms in the western Alabama region, and the causative agent identified was a highly virulent strain of *A. hydrophila* [9]. Fish disease outbreaks are detrimental to achieving sustainability and food security through aquaculture and, hence, are serious challenges to be addressed. *A. hydrophila* is a ubiquitous Gram-negative bacterium with oxidase-positive, facultatively anaerobic, and fermentative phenotypic characteristics. The signs of disease due to *Aeromonas* species include dropsy, ulcers, tail rot, fin rot, and hemorrhagic septicemia, affecting fish species such as carp, tilapia, perch, salmon, catfish, and others [10]. This pathogen has virulence attributes such as the production of cytotoxins and α-β hemolysin toxins and enzymes like phospholipase, proteases, and acetylcholinesterase, which can be toxic to fish cells. The infection caused by *A. hydrophila* has significantly impacted aquaculture in Asian countries such as Thailand, India, the Philippines, and China [11]. To combat the disease, farmers resort to the use of antibiotics; however, their uncontrolled and indiscriminate use has resulted in the development of resistance in pathogens to previously effective antibiotics, leading to therapeutic failure. To overcome the issue of antibiotic resistance in aquaculture systems, vaccination has been considered an effective choice for disease prevention [12]. Given the challenges associated with antibiotic resistance, it is necessary to identify suitable alternatives for disease control, and immune-protective vaccines would be the best [13]. Vaccination and immune stimulation have proven successful in controlling bacterial diseases in fish, and various types of vaccines have been developed to combat bacterial diseases in fish. It is now universally accepted that vaccination has played a pivotal role in the success of large-scale, commercial pisciculture, including the cultivation of salmon, trout, and Indian major carp [14]. However, developing a vaccine can be challenging for emerging pathogens. Probiotic bacteria that produce bacteriocins and other compounds with inhibitory activities against pathogenic bacteria have proved useful in such cases. Over the past two decades, fish vaccines have significantly reduced disease-related losses and resulted in less reliance on antibiotics. Fish vaccines offer advantages over antibiotics as they are naturally biodegradable and do not leave residues in the product or aquatic environment, consequently minimizing the risk of generating antibiotic-resistant strains. While various vaccine options are available, including monovalent, bivalent, multivalent, and DNA vaccines, their long-term immunogenicity in field applications is sometimes questionable due to pathogen variations and host responses. In this scenario, nanovaccines are promising, especially for field applications and long-term sustained protection. Nanotechnology, in collaboration with biotechnology, has made a significant impact on the field of biomedicine [15]. The advancements in this emerging field have also extended into the field of vaccinology, leading to the emergence of nanovaccinology [15,16]. This area holds tremendous potential for advancing vaccine development and delivery methods. It allows the development of novel approaches and makes it possible to reconfigure conventional technologies [17]. In vaccine technology, nanoparticles are used for encapsulating antigens, thus generating nanovaccines that offer targeted protection against infections and diseases by focusing on the site of infection. Nanovaccines, also referred to as the next generation of vaccines, are composed of meticulously designed nanoparticles encapsulated with antigens from pathogens, providing an efficient means to deliver the antigen at the target site and elicit an efficient immune response by safeguarding the antigens from the hostile gastrointestinal environment [18,19]. Nanotechnology has provided a remarkable opportunity to design nanoparticles with diverse sizes, shapes, and surface charges, thereby enhancing their versatility and applicability [20]. Nanovaccines are highly sought after in the aquaculture industry, where they can serve as adjuvants to augment the efficacy of antigens. This review presents recent updates on ongoing research concerning outer membrane protein (OMP)-based vaccines targeting key pathogens that commonly affect the important farmed fish species cultured in India.

## 2. Minimizing *Aeromonas* Infection through Vaccination

Immunostimulants such as OMPs, lipopolysaccharides, extracellular proteins, and S-layers induce immune responses in fish [2,21,22]. Numerous studies have demonstrated the effectiveness of immunostimulants in fish cultivated in warm waters [21,22,23,24]. Experimental studies on different types of vaccines have been shown to successfully elicit immune responses in the host and provide protection upon challenge [25,26]. The choice of vaccines depends on their suitability for the specific fish species and the type of bacterial pathogen. Inactivated vaccines, produced through traditional methods, are widely used for controlling bacterial infections in warm-water fish. Two common types of inactivated vaccines, formalin-treated and heat-killed, are employed to inactivate different bacterial species, which affords a certain degree of protection for the fish [27]. A previous study showed that a formalin-killed vaccine for *A. hydrophila* was more effective in protecting against *A. hydrophila* infection in fish than a heat-killed vaccine [28]. Live-attenuated vaccines are known to elicit a robust immune response in fish that involves both cell-mediated and humoral immune responses, unlike inactivated vaccines that only evoke humoral responses [29,30,31]. A comparative study on determining the efficacy of live-attenuated and formalin-killed vaccines of *A. hydrophila* revealed a higher relative percentage of survival (RPS) among the live-attenuated vaccine (83.7) group compared to the formalin-killed vaccine (37%). Moreover, the expression of immune genes such as IL-1β, IL-10, and Ig-M post vaccination was upregulated at different time points in common carp [32]. *A. hydrophila* is known to produce biofilms enclosed in a self-produced polymeric glycocalyx, which renders them resistant to antibiotics, enzymes, chemicals, and antibodies. Biofilm-based vaccination can overcome the challenges associated with the gastrointestinal destruction of oral vaccines.

### Cellular Components of A. hydrophila Used in Vaccine Development

**Figure 1:**
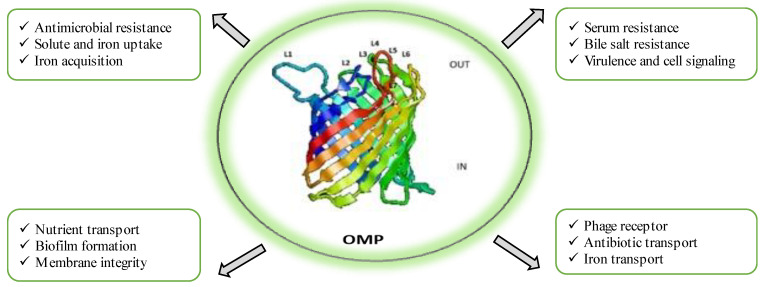
Physiological and pathological responses of outer membrane proteins of *Aeromonas hydrophila*.

OMPs are highly immunogenic due to their exposed epitopes on the cell membrane [8]. In Gram-negative bacteria, OMPs play a crucial role in the initial adherence to host cells, making them potential antigenic entities in vaccine development. OMPs exhibit remarkable diversity in their biological functions, serving as indispensable players in various critical processes such as maintaining structural integrity, facilitating outer membrane biogenesis and maintenance, aiding in nutrient acquisition and transport, mediating ion uptake, promoting cell adhesion, participating in cell signalling, acting as receptors for phages, facilitating waste export, functioning as primary channels for antibiotics and antibiotic efflux, and contributing to biofilm formation, as extensively documented in studies by Liu et al. [33], Xiao et al. [34], Wang et al. [35], Rojas et al. [36], and Acheson et al. [37]. Moreover, OMPs assume pivotal roles in pathogenesis, resistance mechanisms, and the onset of diseases. They do so by serving as dynamic interfaces between the cell and its external environment, scavenging essential nutrients, promoting cell adhesion to other cells, binding to a diverse array of substances, evading host defence mechanisms, and fostering antimicrobial resistance, as highlighted in research by Van der Ley et al. [38], Cowan et al. [39], and Rollauer et al. [40]. Figure 1 illustrates the different physiological and pathological responses of outer membrane proteins. Several studies have demonstrated the efficacy of OMP-based vaccines in inducing immunity against *A. hydrophila* in fish. Vaccination with purified recombinant maltoporin in European eels resulted in protective immunity as a result of increased antibody response, improved lytic activity of the pathogen, and other immunogenic parameters [12]. Likewise, recombinant OmpF immunization in *L. rohita* led to an elevated expression of immune genes and a 44% increase in RPS [41]. Chinese breams vaccinated with recombinant Omp38 exhibited significant protection and reduced histopathological alterations [42]. Vaccination of rohu with r-OmpR and a modified adjuvant showed an upregulation of immune-related genes and an enhanced expression of immune response markers [43]. Lipopolysaccharides (LPSs) derived from Gram-negative bacteria possess immune-modulatory effects and are dominant immune molecules. Injection of LPSs in *Cyprinus carpio* showed improved survival rates due to a concomitant increase in leucocyte count, neutrophils, monocytes, and antibody titre [25]. Crude LPSs from *A. hydrophila* displayed increased immunogenicity in vaccinated fish, regardless of the administration route [44]. The S layer of *A. hydrophila* has been identified as an important antigen conferring protection in common carp [45]. One study on intraperitoneal injection of LPSs, OMPs, and formalin-killed cells in grass carp reported protective efficacy against *A. hydrophila* infection by stimulating strong immune responses and increasing lysozyme activity [45]. Through several studies, it is now well recognized that OMPs and LPSs, as antigenic entities against *A. hydrophila* infection, show promising results in enhancing specific and non-specific immunity in fish, thus proving them as ideal components for vaccine development.

## 3. Types of Nanoparticles Used in Fish Vaccine Preparation

Nanoparticles serve as effective delivery systems that allow for the controlled administration and release of vaccine molecules to achieve sustained prophylaxis and serve as therapeutic delivery agents. They enable targeted delivery to specific cells or tissues, improve bioavailability, enhance the solubilization of hydrophobic drugs, enable controlled release, and protect therapeutic agents, including vaccines, from degradation in the gut when orally administered [19,46]. There are reports on the use of various nanoparticles as adjuvants in vaccine development, and the choice is usually based on compatibility with the host. Commonly employed nanoparticles include polymeric nanoparticles such as alginate, chitosan, poly (lactic-co-glycolic acid) (PLGA), polylactic acid (PLA) dendrimers, and liposomes. However, conjugating the vaccine molecules with suitable nanocarriers becomes crucial in enhancing the properties of a vaccine and optimizing its delivery for effective response [47].

### 3.1. Polymeric Nanoparticles

Polymeric nanoparticles have emerged as promising drug carriers in aquaculture vaccination, attracting considerable attention in recent years. They exhibit desirable properties, including biocompatibility, efficient drug loading, sustained-release capabilities, biodegradability, and improved drug stability, thereby enhancing the potential of aquaculture vaccination [48]. They typically consist of a polymeric shell, often composed of synthetic or natural polymers, encapsulating the desired drug within an inner core [48]. Various polymers, including but not limited to PLGA, PLA, copolymers, and chitosan, are currently used in vaccination trials.

#### 3.1.1. Synthetic Nanoparticles

Among the various types, PLGA- and PLA-based nanoparticles have gained acceptability in vaccine development due to their biodegradable nature, biocompatibility, and sustained-release properties. Encapsulation using these nanoparticles generally does not compromise the structure or function of OMPs, making them suitable for vaccine encapsulation [48]. The copolymer PLGA, composed of lactic acid and glycolic acid, offers control over the degradation rate, the strength of the nanocarrier, and the loading capacity by adjusting the monomeric ratio [49]. Likewise, PLA, another biocompatible and biodegradable polymer, undergoes metabolism into lactic acid monomers within the body [19].

#### 3.1.2. Natural Polymers

These provide distinct advantages in terms of biocompatibility and clearance. Chitosan nanoparticles have been extensively studied and employed in fish vaccine preparations due to their low molecular weight, ease of encapsulation, and sustained-release properties [48,50,51]. Chitosan is non-toxic, biocompatible, biodegradable, and can be easily eliminated from the body without being toxic or leaving a trace [15,52]. Due to their mucoadhesive properties, natural polymers facilitate slow and sustained drug release [15]. For example, chitosan stimulates cytokine production and activates immune cells such as macrophages, natural killer cells, antigen-presenting cells (APCs), and T lymphocytes [53]. Chitosan and alginate are available in various shapes and sizes [2]. In a recent study carried out by Zhang et al. [54] using zebrafish, a biomimetic vaccine delivery system utilizing chitosan nanoparticles demonstrated good biocompatibility and induced an increased production of immune-related antibodies against the spring viremia of carp virus [54].

### 3.2. Metal Nanoparticles

Metals, such as silver, gold, zinc, and titanium, have found extensive application in drug delivery for warm-water fish. These metal nanoparticles offer distinct advantages compared to polymers, which include their large surface area, tuneable size, precise synthesis control, efficient drug loading, and trackability. Among these advantages, precise drug delivery using metal nanoparticles has garnered significant attention. A recent study by Zhang et al. [55] demonstrated the use of mesoporous silica nanoparticles (MSNs) in delivering the dihydrolipoamide dehydrogenase antigen against *Vibrio alginolyticus* in yellow croaker fish, and the results were promising, particularly for pH-mediated drug release [55]. Zinc oxide nanoparticles exhibit antibacterial activity by causing damage to the cell membrane and cytoplasmic content, leading to bacteria cell death. In fish prophylaxis, zinc nanoparticles have shown inhibitory effects against the growth of *A. hydrophila*. Likewise, titanium dioxide nanoparticles have been shown to enhance the fish immune response and exhibit antibacterial activity [48].

### 3.3. Liposomes

These are biologically inert, non-toxic, and biodegradable vesicles composed of hydrophobic and hydrophilic molecules that mimic the structure of lipid bilayers and have been used extensively in vaccine development [18,56]. Immunostimulating complexes (ISCOMs), which consist of cholesterol, phospholipids, quillaia, and saponins, are known for their immune-response-stimulating properties and, therefore, are commonly used as adjuvants to provide extended protection [15,57]. ISCOMs can induce both cellular and antibody responses. Virus-like particles (VLPs) containing capsid proteins are also utilized in vaccine formulations to combat outbreaks caused by pathogens [18]. VLPs elicit immune responses even without the use of adjuvants. Additionally, the properties of VLPs, being replication-incompetent and devoid of viral genetic material, ensure their safety [58,59]. VLPs, particularly virus protein-based liposomes, are widely employed because they stimulate robust immune responses [60].

### 3.4. Emulsions

**Figure 2:**
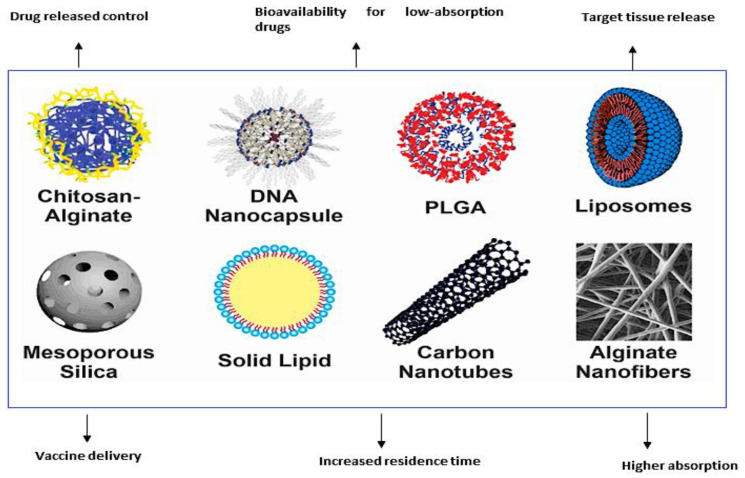
Different nanoparticles used in vaccine delivery. (Reproduced and reprinted with permission from Fajardo et al., [61], copyright 2022 with permission from KeAi, https://doi.org/10.1016/j.aaf.2021.12.006. (accessed on 10 March 2023). https://creativecommons.org/licenses/by/4.0/.)

Emulsions represent another form of nanomaterials utilized in vaccination and exist in two forms: oil-in-water and water-in-oil. They comprise droplets with sizes ranging from 50 nm to 600 nm and facilitate the desired site-specific vaccine delivery by achieving targeted delivery of antigens to dendritic cells [20]. However, they are thermodynamically unstable. They can be utilized by mixing with antigens to deliver vaccines to the intended site [20]. Figure 2 illustrates the different nanomaterials employed in vaccination.

## 4. Highlights of the Fish Immune System

The immune system of fish is highly intricate. It consists of various specialized lymphatic tissues, comprising the mucosal-associated lymphatic tissue (MALT), gut-associated lymphatic tissue (GALT), skin-associated lymphatic tissue (SALT), and gill-associated lymphatic tissue (GIALT) [62,63]. Each tissue exhibits a specific immune response mediated by immune cells and antibodies in the bloodstream [56]. The effectiveness and type of the immune response depend on the route of vaccination as different tissues are involved [15]. When nanovaccines encounter antigen-presenting cells (APCs) like macrophages, phagocytic B-cells, and dendritic-like cells, they are engulfed and internalized. Apart from macrophages, phagocytic B-cells [64,65] and dendritic-like cells have been reported in fish and have been shown to play a crucial role in initiating and coordinating these immune responses [66,67]. Upon engulfing nanovaccines containing the OMP antigen, the APCs undergo maturation and present the antigen molecules on the surface of major histocompatibility complex (MHC) class I and MHC class II molecules that present the antigenic peptides to B and T lymphocytes to activate the adaptive immune system. This process accompanies the secretion of signalling molecules and morphological changes in APCs [68]. Antigens are internalized by APCs through either the endocytic or non-endocytic pathway. In the endocytic pathway, foreign particles are engulfed through phagocytosis, and proteolytic enzymes aid in the degradation of antigens. The resulting degraded peptides are then presented on MHC class I and II molecules and recognized by CD4 and CD8 T cells. These stimulate B and T cells to produce antibodies and activate T cell responses. In the non-endocytic pathway, antigens are processed by the proteasome complex, and the resulting peptides are presented on MHC class I molecules. These peptides are recognized by CD8 T cells, which exhibit cytotoxic activity against infected cells [69].

## 5. Nanoparticle-Mediated Antigen Delivery and Mode of Action

**Figure 3:**
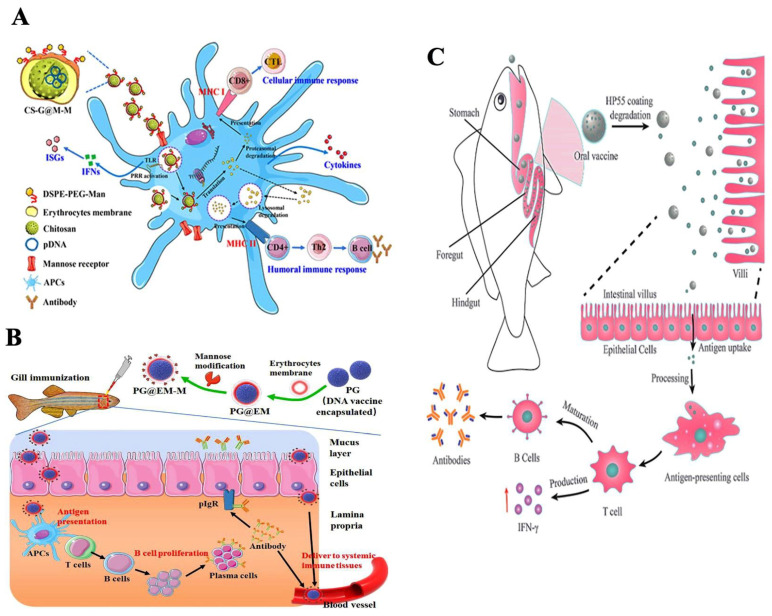
Different nanoparticle-based fish vaccines and their immune responses: (**A**) Biomimetic cell-derived nanoparticles with surface mannose as vaccine delivery platform against viral teleost fish disease (reproduced and reprinted with permission from ACS from Zhang et al. [54], Copyright 2020 with permission from ACS Biomater. https://doi.org/10.1021/acsbiomaterials.0c01302 (accessed on 12 March 2023) https://creativecommons.org/licenses/by/4.0/). (**B**) Mucosal delivery of mannose-functionalized biomimetic nanoparticles via the branchial route induces robust mucosal and systemic immune responses against viral fish diseases (reproduced and reprinted with permission from Zhang et al. [76], copyright 2022 with a permission from Aquaculture. https://doi.org/10.1016/j.aquaculture.2021.737329 (accessed on 12 March 2023) https://creativecommons.org/licenses/by/4.0/). (**C**) pH-controlled release of antigens using a mesoporous silica nanoparticle delivery system for developing an oral fish vaccine (reproduced and reprinted with permission from Zhang et al. [55], copyright 2021 with permission from Frontiers in Immunology. https://doi.org/10.3389/fimmu.2021.644396 (accessed on 12 March 2023) https://creativecommons.org/licenses/by/4.0/).

Nanovaccines are known to elicit both innate and adaptive immune responses in fish. Nanoparticles encapsulated with specific antigens induce specific cellular and humoral immune responses. The adaptive immune system, mediated by lymphocytes, encompasses three domains: humoral immunity, cell-mediated immunity, and immunological memory. In fish, humoral immunity involves the production of immunoglobulin (Ig) types IgM, IgD, and IgT by B cells [70]. The cellular immune system relies on cytotoxic T-lymphocytes, which are crucial in recognizing and combating pathogens. Adaptive immunity recognizes pathogens through molecules generated by somatic pathways, followed by humoral and cellular responses mediated by B and T lymphocytes [70]. APCs are responsible for antigen processing and presentation via MHC class II or MHC class I molecules, including cross-presentation. The interaction of nanoparticles with dendritic cells results in the expression of co-stimulatory molecules, which is dose-dependent. The characteristics of nanoparticles, such as their size and polymer composition, significantly influence dendritic cell maturation. Additionally, surface interactions between nanoparticles and antigen-presenting cells affect dendritic cell uptake. Dendritic cells preferentially take up small particles (20–200 nm), while macrophages prefer larger particles (0.5–5 µm). The shape and surface charge of nanoparticles are crucial in determining the physicochemical factors and interactions between nanoparticles and antigen-presenting cells. Cationic particles, due to the anionic nature of cell membranes, induce phagocytic activity in antigen-presenting cells. Furthermore, nanoparticle shape influences phagocytosis by macrophages, with hydrophobic particles causing a more robust immune response than hydrophilic ones [68]. When administered, nanoparticles can elicit various innate immune responses, but they are not inherently immunogenic unless conjugated with an antigen. Different nanoparticles can activate pattern recognition receptors, induce cytotoxic T lymphocyte responses, stimulate T-helper (Th) cells, promote cytokine production, activate B cells, and trigger antibody production [71]. The size of nanoparticles may also play a role in the type of immune response generated, with antigen-presenting cells preferentially taking up particles based on their size [72]. Several studies suggest that small particles induce stronger immune response than large-sized ones [73]. Nanoparticles can deliver antigens through two methods: transient delivery and co-ingestion delivery. The attachment of antigens to nanoparticles can be achieved through physical absorption or more complex methods, such as chemical conjugation or encapsulation [74]. Physical absorption relies on weak interactions such as basic charge or hydrophobic interactions between the antigen and the nanoparticle [75]. Strong interactions are achieved through chemical conjugation or encapsulation, where antigens are chemically cross-linked to the nanoparticle surface and subsequently released inside cells [75]. Nanovaccines facilitate antigen delivery to lymphoid organs through direct drainage or by activating APCs to induce an immune response. Small-sized nanoparticles can efficiently navigate through the extracellular matrix and reach the lymphoid organs, and, on reaching the site, APCs are activated. Recent studies have demonstrated the effective targeting of APCs by the developed cPG@EM-M nanovaccine, particularly through mucosal delivery via the branchial route, leading to robust mucosal and systemic immune responses [76]. Figure 3A Illustrates a biomimetic vaccine delivery platform that encapsulates a chitosan-loaded DNA vaccine within teleost erythrocyte membranes modified with mannose. The developed CS-G@M-M nanovaccine delivery platform has demonstrated excellent biocompatibility in vivo and in vitro. The CS-G@M-M vaccine exhibits enhanced uptake by antigen-presenting cells (APCs) and a notable increase in its accumulation within immune tissues, including the spleen, kidney, and hindgut. Remarkably, the CS-G@M-M nanovaccine demonstrates prolonged immunoprotection efficacy, effectively safeguarding zebrafish from challenges posed by the spring viremia of carp virus (SVCV). This innovative design of smart teleost erythrocyte-membrane-coated nanoparticles, known for their inherent biocompatibility, holds great promise in inducing robust adaptive immune responses to prevent viral diseases in fish [54]. In Figure 3B, we observe a pioneering biomimetic nanovaccine developed by encapsulating a poly (D, L-lactide-co-glycolide)-based DNA vaccine within teleost erythrocyte membranes modified with mannose. To assess local and systemic immunity, zebrafish, employed as a model organism, were immunized with the PG@EM-M nanovaccine via the branchial route. SVCV served as the model virus for the challenge study. This research introduces an innovative concept of nanoparticles that mimic erythrocytes for mucosal immunization. This development can enhance vaccine efficacy and refine the design of mucosal vaccines for application in aquaculture [76]. Figure 3C shows that the dihydrolipoamide dehydrogenase (DLDH) antigens from *V. alginolyticus* were encapsulated within mesoporous silica nanoparticles (MSNs) to create an effective vaccine delivery system. A coating of hydroxypropyl methylcellulose phthalate (HP55) was used to safeguard the immunogen. The vaccine’s immunogenicity and protective efficacy were assessed in large yellow croakers through oral administration. The in vivo administration of the nanoparticle-based vaccine induced both innate and adaptive immune responses, effectively providing robust protection against *V. alginolyticus* infection [55].

Nano-oral vaccines have also been shown to stimulate long-lasting innate and adaptive immunity by loading nanoparticles with protective antigens and enriching the data on innate immune factors of fish [54]. The route of administration and the biological environment can affect nanoparticle drainage to lymphoid organs, particularly for large molecules encountered by APCs. Following successful antigen delivery, nanoparticle clearance occurs through renal clearance or degradation by the immune system, thereby preventing accumulation in different tissues and minimizing adverse effects.

## 6. Routes of Vaccine Administration

Vaccines can be administered to fish through various routes, viz. parenteral (intramuscular or intraperitoneal injection), immersion (bath or dip-vaccination), and oral administration [77]. The chosen route of vaccine administration can influence the level of protection induced and the immunological response elicited against the targeted pathogen [78]. The effectiveness of the vaccine delivery method depends on factors such as the route of administration, pathogen type, fish life stage, and water parameters during the immunization process [79]. While nanoparticle-based vaccines have been extensively studied for response through the oral and parenteral routes, there is a research gap in understanding their applications in nasal, buccal, and topical delivery systems [47,80]. A list of vaccines designed to prevent *A. hydrophila* infection is provided in Table 1. In the context of vaccination in fish, various routes of administration are employed, each with its own advantages and limitations. 

### 6.1. Parenteral Vaccination

#### 6.1.1. Intramuscular Route

The intramuscular route triggers cellular and humoral immune response, facilitated by APCs in the muscle tissue. However, these cells cannot directly activate T cells due to the absence of MHC II expression [68,99,100]. Intramuscular injection is an effective method, providing long-lasting protection [100]. However, it can be challenging, time-consuming, and costly for farmers, particularly when dealing with small fish (<20 g) that are more susceptible to diseases [101]. Several studies have successfully demonstrated the generation of acquired and enhanced innate immunity in intramuscularly immunized fish [2,102,103,104,105,106,107,108].

#### 6.1.2. Subcutaneous Route

The subcutaneous route allows antigen drainage from the injection site to lymphoid organs. Small particles can enter lymphatic vessels, while the large-sized ones undergo phagocytosis by APCs before passage into lymphoid organs [109,110,111].

### 6.2. Mucosal Vaccination

#### 6.2.1. Oral Vaccination

This technique protects antigens from degradation and is convenient for mass vaccination, particularly through feed in fish farms. However, determining the precise antigen dose and absorption mechanism in the gastrointestinal tract can be challenging [112]. Primary oral vaccination may not induce a robust immune response, but an oral booster dose can lead to a strong secondary anamnestic response [113]. The efficacy of oral vaccines depends on various factors, including the nature of the antigen and the dose regimen and formulation [114]. Biodegradable carrier-based vaccines have demonstrated protective immunity against *A. hydrophila* when administered orally [115].

#### 6.2.2. Immersion Vaccination

This method involves the exposure of fish to a vaccine in water, which allows for a stress-free administration but has drawbacks such as requiring a large amount of vaccine, providing low vaccine protection, and limited antigen uptake by the skin and gills. Different immersion methods include direct immersion (DI), hyperosmotic infiltration (HI), and spray. HI fell out of use due to stress concerns, while DI has effectively conferred protection [116,117]. Novel immersion methods, such as combined immersion/puncture immunization or ultrasound-assisted delivery, have also been explored [118,119].

#### 6.2.3. Intradermal Vaccination

This involves injecting the vaccine into the outermost skin layer, reaching the epidermis. Nanovaccine-based intradermal administration has been shown to induce the maturation of APCs and enhance the immune response [120].

## 7. Nanovaccines against *Aeromonas* Infection in Fish

**Figure 4:**
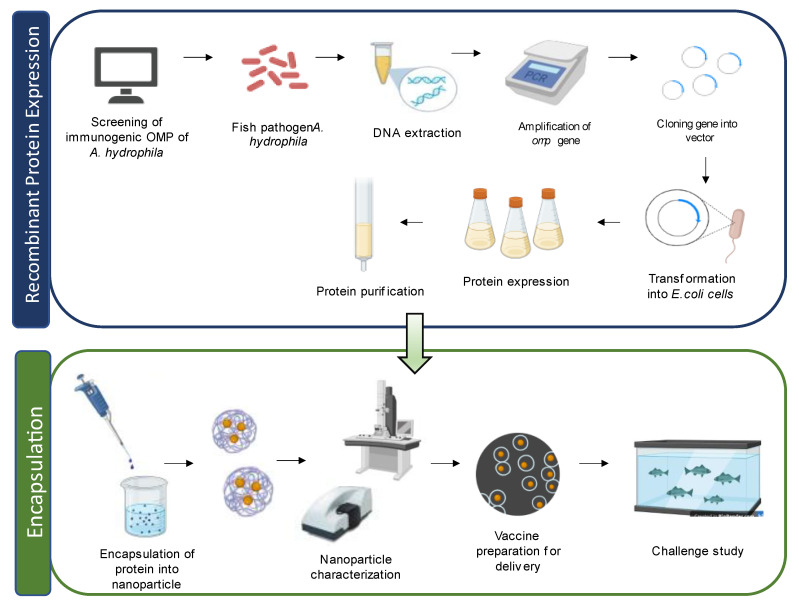
Schematic diagram of recombinant outer membrane proteins (OMPs) in nanovaccine preparation for fish.

In a study by Liu et al. [33], grass carp treated with an OMP-loaded PLGA nanovaccine showed a higher immune response against *A. hydrophila* infection compared to carp treated with a DNA vaccine encapsulated with single-walled carbon nanotubes. This nanovaccine induced the expression of various immune-related genes, such as interferon I (IFN-I), tumor necrosis factor (TNFα), C-reactive protein (CRP), interleukin (IL-8), IgM, MHC I, and CD8 in the kidney of grass carp. Another study by Dash et al. [86] demonstrated the effectiveness of OMP conjugated with PLGA and PLA nanoparticles as vaccines in *L. rohita*, providing enhanced protection against *A. hydrophila* infection. Both PLGA/PLA OMP nanoparticles exhibited promising results by activating the innate immune response in fish without any adverse effects. They also showed increased agglutinating titre, hemolytic activity, specific antibody titre, and RPS upon challenge with *A. hydrophila*. Guo et al. [83] tested specific protein-encapsulated SWCNTs in zebrafish and found improved antigen delivery and prolonged immune response. Nanoparticle-encapsulated aerA vaccines demonstrated enhanced antibody production and an increased survival rate compared to the free-aerA-injected group against *A. hydrophila* infection. Vijayakumar et al. [121] explored the use of fucoidan-coated gold nanoparticles (FU-Au) for antimicrobial therapy in controlling *A. hydrophila* infection in *Oreochromis mossambicus*. Administration of FU-Au nanoparticles resulted in an increased survival rate and improved recovery from bacterial infection. Likewise, Dubey et al. [90] demonstrated the protective immunity conferred by oral administration of OmpW using PLGA nanoparticles in *Labeo rohita* against *Aeromonas* infection, suggesting its potential for prophylactic application in aquaculture farms. Comprehensive lists of different types and kinds of nanovaccines against *A. hydrophila* infection in fish are summarised in Table 2 and Table 3, respectively. Figure 4 illustrate the preparation of the nanovaccine using OMPs.

## 8. Immunological Memory in Teleost Fish

Vaccination is effective as a prophylactic strategy against infectious diseases in the host species with proven adaptive immunity. Antibodies generated in response to vaccination are considered to be the most reliable correlate of protective immunity in fish [126], and the humoral immune response is similar to observations made in humans [127,128]. Several challenge studies have reported a decrease in the level of protection corresponding with a decline in antibody titres. This unequivocally suggests that preformed antibodies prior to the challenge of fish are the most important in protection [129,130,131]. Antigen (pathogen) neutralization during challenge by antibodies developed during vaccination is an important protection mechanism. The anamnestic response observed following booster administration in fish has been shown to increase antibody responses after the second and third vaccination doses [132,133]. Ma et al. [134] reported a secondary response in rainbow trout (*Oncorhynchus mykiss*) immunized with trinitrophenylhapten (TNP) covalently conjugated to bacterial lipopolysaccharides (LPSs), which served as the TNP-LPS antigen. The secondary response was significantly prolonged compared with the primary response. They observed that antibody-secreting cells (ASCs), both plasma cells and plasma blasts, quantitatively paralleled antibody production with ASC skewing to the hematopoietic anterior kidney. However, they also observed an enhanced antigen-inducible response indicative of the memory pool that skewed into the peripheral blood and spleen. They noted that this pattern of memory response parallels observations seen in mammals even though the organization of the immune system differs between fish and mammals. Kaattari et al. [135] proposed an alternative model of the functional differentiation and regional distribution of ASCs in rainbow trout that are essential for developing immunological memory and aquaculture vaccines. Karunasagar et al. [136] investigated the impact of thymectomy on the humoral response of rohu, *Labeo rohita*, to an *A. hydrophila* vaccine. The results of the study revealed that non-thymectomized fish were fully protected when challenged with the pathogen, whereas the partially thymectomized fish exhibited limited protection. The bacterial cell vaccine possessed both T-dependent and T-independent antigens. Results of this showed a notable abundance of thymus-independent (TI) antigens, such as lipopolysaccharide. Millar et al. [137] demonstrated that an effective response to TI antigens required the presence of both B cells and macrophages.

Conversely, for a response to thymus-dependent (TD) antigens, B cells, macrophages, and T helper cells were essential. Building upon this knowledge, Avtalion et al. [138] suggested that T cell function played a crucial role in developing memory cells in fish. Stosik et al. [139] conducted studies on teleostei that showed evidence supporting the existence of immunological memory mediated by both T and B cells, resulting in a secondary response that was more robust and rapid than the primary response. This experimental study demonstrated how re-exposure to the same antigen triggered a reaction from the previously activated specific cells. These cells possess a unique characteristic of retaining the immunological memory acquired during the initial encounter with the antigen. This state of heightened immune activity can also be interpreted as a consequence of altered behaviour of the immune system brought about by genetic alterations. Importantly, these genetic changes appear to persist independent of antigen presence. Furthermore, the findings suggest an alternative mechanism for sustaining a lowered level of immune response, either guided by or dependent on recurrent exposure to the same antigen over time. Additionally, the concept of innate immune memory response involves epigenetic reprogramming of myeloid cells, specifically macrophages and NK cells.

Magadan et al. [140], in their investigation, focused on elucidating the origin of public memory B cell clones in fish upon antiviral vaccination. The results of their study unveiled a substantial expansion of public clonotypes post vaccination, characterized by numerous VDJ junctions that exhibited minimal differences, typically only one or two amino acids, while still possessing similar functional properties. This phenomenon underscored a convergent response among the vaccinated fish. Consequently, the collective memory antibody response to the virus in the fish emerged as a product of multiple factors viz. recombination bias, selection process affecting the formation of the pre-vaccination repertoire, and the convergent selection of clonotypes that functionally resembled each other during the response. Furthermore, the study shed light on the distinct structures and inter-individual variation in the naive repertoires of IgM and IgT due to inherent selection bias. The memory pool of ASC described above is expected to play a crucial role in conferring protective immunity in fish vaccinated against *A. hydrophila,* which would be enhanced with adjuvants when using nanovaccines for sustained release. 

## 9. Advantages and Disadvantages of the Application of Nanovaccine

### 9.1. Advantages

The biodegradability and biocompatibility properties of nanoparticles make them a suitable candidate as an adjuvant in nanovaccine applications against several bacterial infections in aquaculture. Nanoparticles protect the antigen from degradation and retain the characteristic shape of the vaccine candidate. They can be adjusted to mimic the characteristics of the pathogen and can drain into lymphoid organs. They are easily internalized by APCs and used to present antigenic peptides to adaptive immune system cells via the MHC-I and II molecules. The nanoparticle ensures that the vaccine candidate retains its original size and charge. It also plays a major role in the retention and biodistribution of particles in lymphoid organs like the headkidney and spleen in fish. Administering nanoparticles effectively protects against fish pathogens and helps generate protective immunity against infection. In addition, nanoparticles aid in cellular uptake mechanisms like phagocytosis, macropinocytosis, and endocytosis [68]. A large surface area ratio helps in target-specific delivery via conjugation with receptor–ligands or antibodies. Synthesis of a nanoparticle is feasible compared to any other adjuvant, with the advantages that it does not require any cold chain for preservation, is thermostable, and can potentially reduce the vaccine dose and side effects while parallelly increasing antigen delivery efficiency coupled with enhanced immune protection [122]. This enhanced immunogenicity of the weak antigen offers an advantage over conventional adjuvant approaches through controlled release kinetics, prolonged stability, and targeted delivery. It also can be used as a standalone entity, as an adjuvant to stimulate an immune response. Its conjugation with vaccines allows gastrointestinal stability, which is central to oral vaccine development [122,141]. The role of PLGA and PLA nanoparticle-encapsulated *A. hydrophila* OMPs in increased immune response has been reported. Nanotubes are useful antigen carriers and can translocate the bioactive molecule to the antigen-presenting cells [13]. Even single-walled carbon nanotubes enhance the immune protection afforded by DNA vaccines and act as the delivery vehicle for recombinant proteins targeting specific pathogens, as observed through studies conducted in common carp, zebrafish, and rohu [83].

### 9.2. Disadvantages

Notwithstanding the advantages associated with nanoparticles, it is important to note that problems associated with their use need to be addressed. Producing non-aggregated and stable nanoparticles with desirable properties and consistency is a major challenge. The gap in knowledge regarding the role of the physical and chemical properties of nanoparticles, their distribution, target, and biosystem interactions needs refinement through further research [48,142]. Due to their small size, nanoparticles need to be handled with dexterity with thorough knowledge about the distribution, process, and difficulty in formulation for field applications. Though the most studied, PLGA- and PLA-based nanoparticle delivery systems have drawbacks, including high initial burst, incomplete release, and protein instability coupled with safety issues pertaining to the vaccine. Once they are released into the environment, the possibility of their interaction with their surroundings could result in bioaccumulation [67]. Nanotoxicity in aquatic animals due to the conjugation of a biomolecule with nanoparticles concentrated in the gills, liver, and brain tissues for long periods is irreversible and results in oxidative stress. This primarily affects nanoparticle-induced immunotoxicity in fish, their phagocytic activity, and cell-mediated immunity. This toxicity affects the lysosomal distribution, alters phagocytosis, and changes the function of phagocytic cells [143].

## 10. Conclusions

In conclusion, nanotechnology-based vaccines hold great promise in general, controlling and preventing bacterial infection in fish culture systems and *A. hydrophila* infection in particular. In order to qualify for effective field use, these vaccines should meet important criteria, including safety for the environment and handlers, ease of administration, strong immune potency, minimal side effects, and low toxicity to aquatic systems and fish. Oral delivery of nanovaccines offers significant advantages in controlling *A. hydrophila* infection. However, further research is needed to explore and optimize ideal nanoparticles such as liposome nanotubes, dendrimers, and nanocapsules to effectively deliver fish vaccines. The investigation of nanoparticle toxicity, their mechanisms in biosystems, and the bioavailability of nanoparticles require comprehensive understanding. Additionally, studies focusing on the physicochemical properties, distribution, stability, and efficacy of nanoparticles for administration to fish are crucial. Identifying ways to minimize the side effects of nanovaccines while providing long-term immunity is a significant challenge in fish vaccination. Given the economic feasibility and efficacy of nanovaccines, their utilization in aquaculture, particularly in developing countries, is highly recommended. Continued basic research is necessary to advance the field of fish vaccination and to improve the overall effectiveness of nanotechnology-based vaccines.

## Figures and Tables

**Table 1:** Different vaccines to prevent *A. hydrophila* infection in fish.

Antigenic Entity	Model Fish Species	Country	Administration Route	Reported Efficiency in RPS	Reference
OmpII-U-A	Eel	China	IP	66.7	[81]
OMP	European eel	China	IP	83.4	[82]
Iron-related recombinant protein	Zebrafish	China	IP	66.67	[83]
Oral yeast-based DNA vaccine (OmpG)	Goldfish	China	IP	46.7	[84]
OmpF	Rohu	India	IP	-	[41]
Tdr	Catfish	USA	IP	95.59	[85]
Tbpa	Catfish	USA	IP	47.89	[85]
OmpR	Rohu	India	IP	-	[86]
Maltoporin	European eel	China	IP	75	[12]
ISKNC ORF 086 protein + OmpA	Chinese perch	China	IP	73.35	[87]
Live-attenuated vaccine	Carp	China	IP	83.7	[88]
DNA vaccine + carbon nanotubes (*aerA* gene)	Grass carp	China	IM	-	[89]
OmpW + PLGA	Rohu	India	Oral	79.9	[90]
OMP fimA	Catfish	USA	IP	59.83	[10]
Fim	Catfish	USA	IP	95.41	[10]
MrfG	Catfish	USA	IP	85.72	[10]
Fimomp	Catfish	USA	IP	75.01	[10]
OMP + PLGA	Rohu	India	IP	-	[11]
OmpR	Rohu	India	IP	52	[43]
Omp38	Chines breams	China	IP	57	[42]
Omp48	Rohu	India	IM	69	[91]
OMP Aha1	Common carp	India	IP	52	[92]
OmpW	Common carp	India	IP	71	[93]
FK, Vaccine	Rainbow trout	Iran	IP	67	[94]
HK	Rainbow trout	Iran	IP	84	[94]
LPS	Rainbow trout	Iran	IP	34	[94]
LPS	Grass carp	China	IP	83.35	[44]
OMP	Grass carp	China	IP	72.2	[44]
FK	Grass carp	China	IP	55.6	[44]
S-layer vaccine	Common carp	Japan	IP	-	[45]
OMP + PLGA	Rohu	India	IP	-	[95]
OmpTS	Rohu	India	IP	-	[24]
OMP	Goldfish	India	IP	50	[24]
Aero A live vaccine	Rainbow trout	Spain	IP	-	[96]
LPS	Carp	India	IP	-	[25]
Major adhesion (Aha1)	Carp	Singapore	IP	87.5	[97]
OMP	Goldfish	Japan	IP	-	[98]

LPS: lipopolysaccharide, OMP: outer membrane protein, FK: formalin killed, HK: heat killed, IM: intramuscular, IP: intraperitoneal. RPS: relative percentage survival.

**Table 2:** Different types of nanoparticles.

Different Types of Nanoparticles	Advantage	Disadvantage	References
(1) Polymeric nanoparticle (a) Synthetic nanoparticle (b) Natural nanoparticle	▪ Biodegradable ▪ Surface modification is easy ▪ Targeted antigen delivery ▪ Better immunogenicity	▪ Low antigen-loading capacity ▪ Antigen degradation ▪ Premature release of antigen	[15,18,19,20,54,122,123]
(2) Inorganic nanoparticle	▪ Easy to prepare ▪ Sustained release	▪ Low biodegradability ▪ Low aqueous solubility	[19,123]
(3) Virus-like particle	▪ High gastrointestinal stability	▪ Lack of reproducibility	[18,123]
(4) ISCOMs	▪ Antigen encapsulation is easy	▪ Antigen incorporation is difficult ▪ Lack of reproducibility	[18,123]
(5) Emulsion	▪ Self-adjuvant property	▪ Thermodynamically unstable	[18]

**Table 3:** Different nanovaccines against *A. hydrophila* infection.

Antigenic Entity	Pathogen	Adjuvant/Carrier System	Fish Model	Route of Administration	RPS	Reference
Recombinant protein	*A. hydrophila*	SWCNT	*Danio rerio*	IP	94	[83]
DNA vaccine(aerA)	*A. hydrophila*	SWCNT	*Ctenopharyngodon idella*	IM	83	[13]
rOmpW	*A. hydrophila*	PLGA	*Labeo rohita*	Oral	79.9	[90]
rOmp	*A. hydrophila*	PLGA PLA	*Labeo rohita*	IP	80 75	[11]
MOMP	*A. hydrophila*	ISCOMs	*Anguillia anguillia*	IP	80	[124]
S-layer protein	*A. hydrophila*	Calcium phosphate	*Labeo rohita*	IP	100	[125]
aerA	*A. hydrophila*	OCMCS-hyaluronic acid	*Cyprinous carpio*	Oral	ND	[13]

IM: intramuscular, IP: intraperitoneal. RPS: relative percentage survival. PMMMA: poly [(methyl methacrylate)-co-(methyl acrylate)-co-(methacrylic acid)], PLA: poly (lactic acid), SWCNT: single-walled carbon nanotubes, ISCOMs: immunostimulating complexes, PLGA: poly (lactic-co-glycolic acid), OCMCS: oleoyl-carboxymethyl-chitosan, ND: not determined.

## Data Availability

Not applicable.
